# Supplementary material for: Mesopelagic fishes dominate otolith record of past two millennia in the Santa Barbara Basin
Source: Nat Commun. 2019 Oct 8;10:4564. doi: 10.1038/s41467-019-12600-z (PMC6783546; doi:10.1038/s41467-019-12600-z)
Supplement: Supplementary file 1 — Supplementary Information [file 41467_2019_12600_MOESM1_ESM.pdf]

Supplementary Information

Mesopelagic fishes dominate otolith record of past two millennia in the Santa Barbara Basin

Jones and Checkley

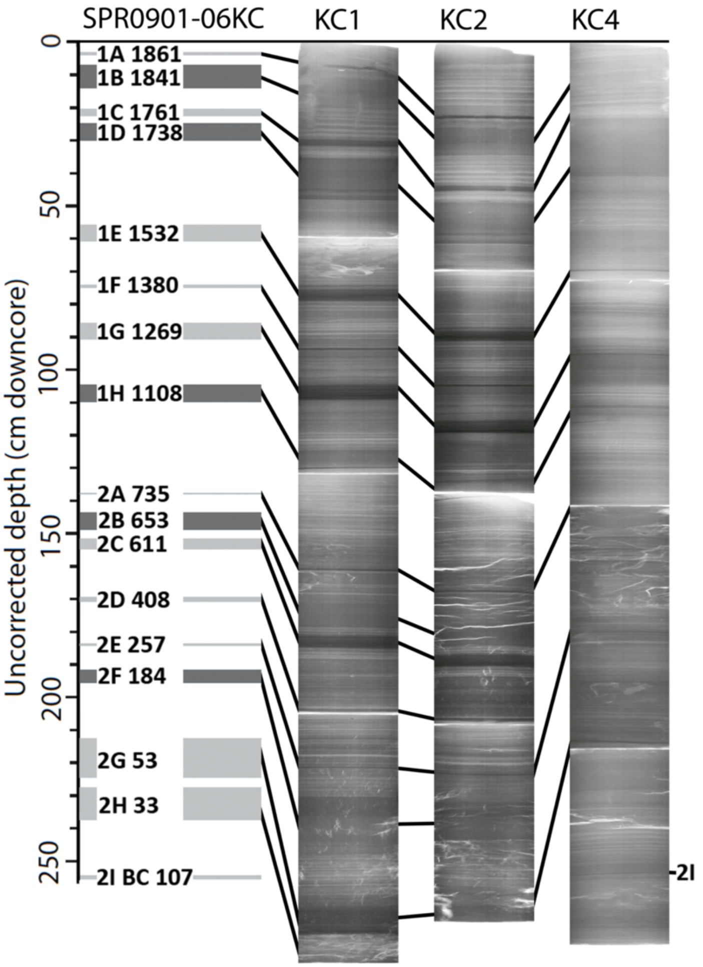

Supplementary Figure 1. X-rays of Kasten cores used to develop chronology. Prominent near-instantaneous events are labeled using the notation of Hendy *et al*. (ref. 1). Distinct events were cross-dated across cores and with core SPR0901-06KC to develop the chronology for cores KC1, KC2, and KC4 (BC1 not shown).

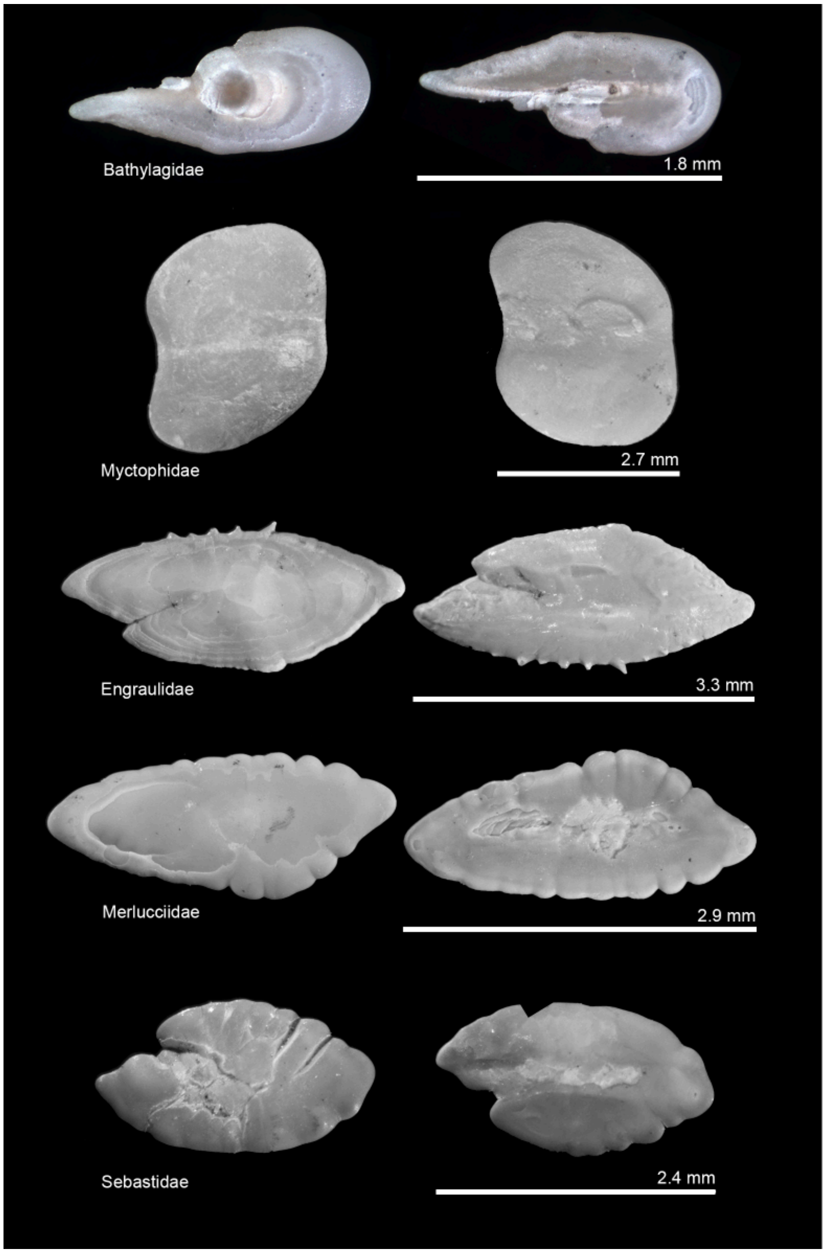

Supplementary Figure 2. Otoliths representative of families of fishes common in the Santa Barbara Basin. Left images show side without sulcus acusticus (medial surface groove). Right images show side with sulcus acusticus. Otoliths are from whole fishes of known species. A catalogue of otoliths from representative species in and near the Santa Barbara Basin is in ref. 2.

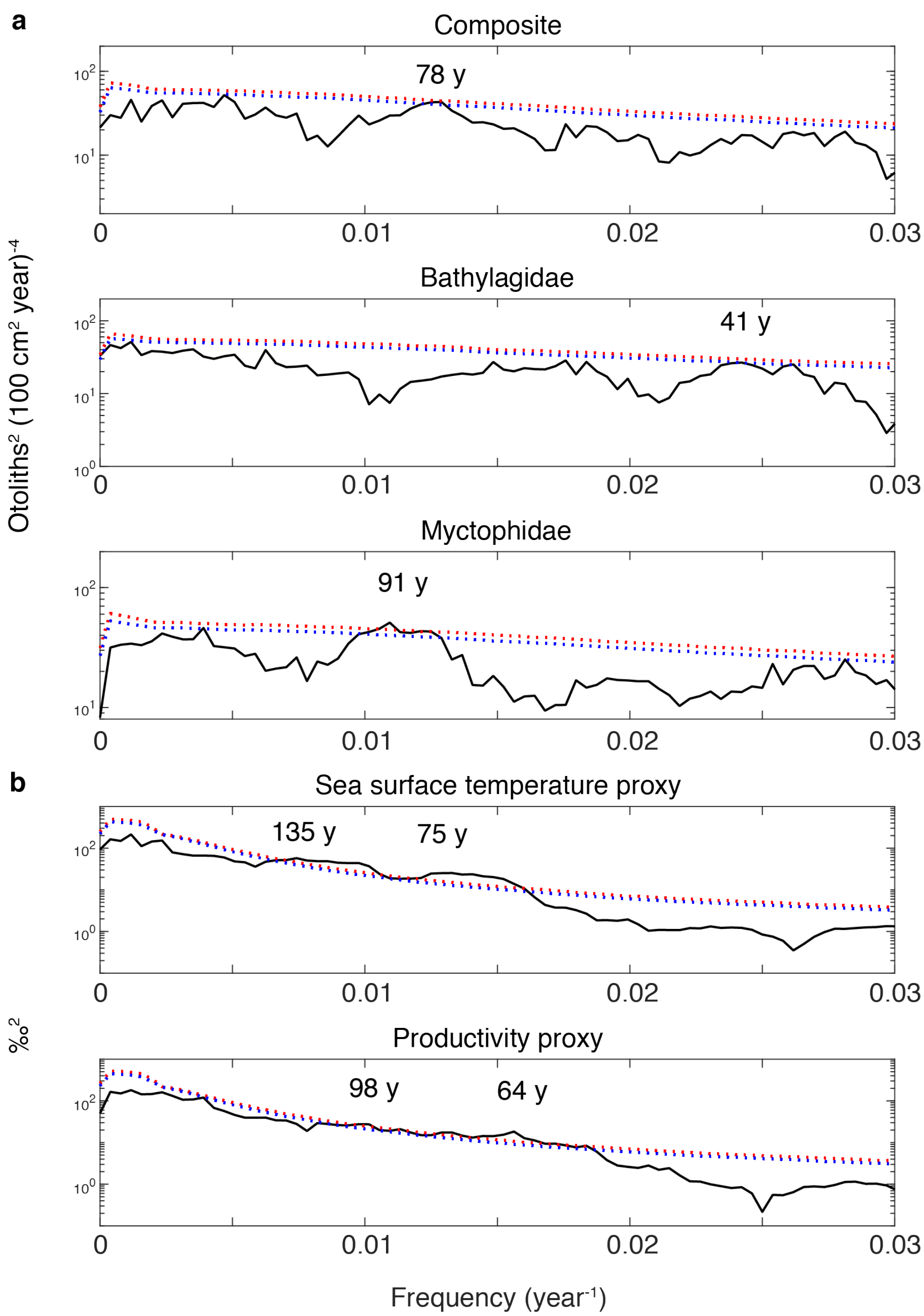

Supplementary Figure 3. Global power spectra for otolith deposition rate and environmental proxies. a, Global power spectra for otolith deposition rate of Composite (all classified otoliths, $n_{otoliths}$ = 1188), Bathylagidae ($n_{otoliths}$ = 367), and Myctophidae ($n_{otoliths}$ = 413). **b**, Global power spectra for proxies of sea surface temperature and primary productivity (10-y bins, n_{bins} = 188). No filters used. Dotted lines are confidence limits (red, 95%; blue, 90%; two-sided) computed assuming a red-noise background with first-order autocorrelation. Periods of major peaks exceeding 95% are labeled. See Methods for details. Source data are provided as a Source Data file.

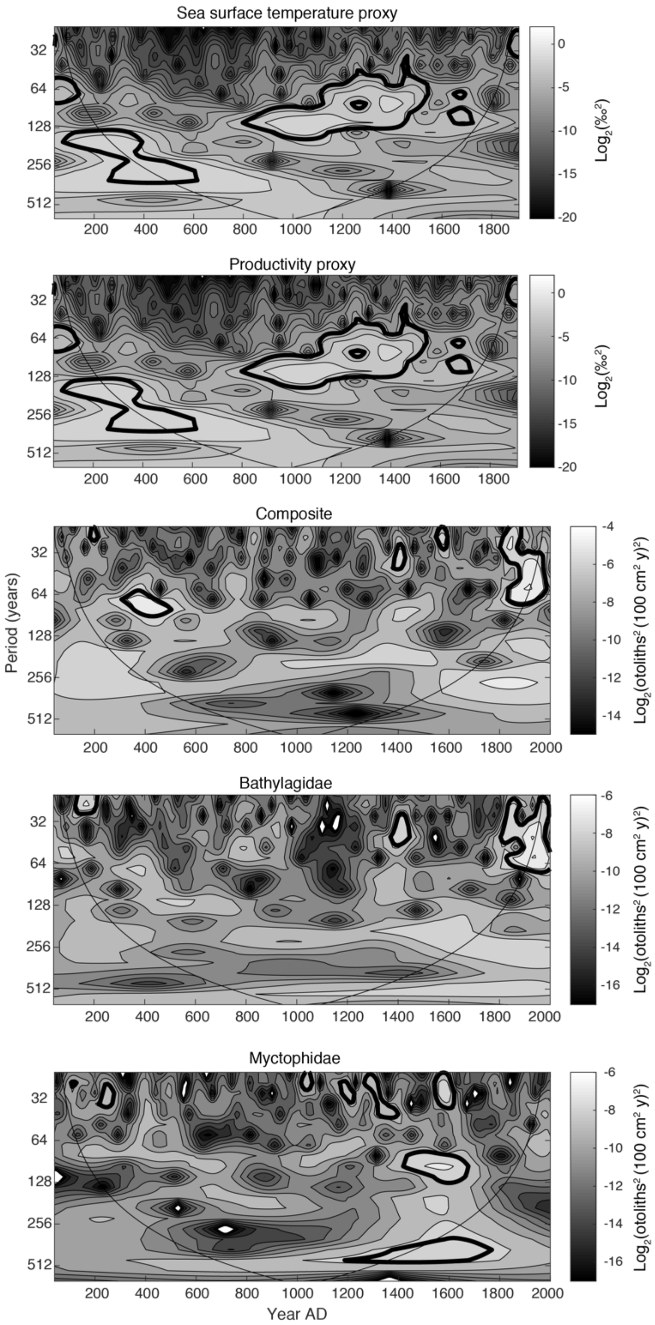

Supplementary Figure 4. Wavelet power spectra for otolith deposition rate and environmental proxies. a, Wavelet power spectra for otolith deposition rate (ODR) for Composite (all classified otoliths, $n_{otoliths}$ = 1188), Bathylagidae ($n_{otoliths}$ = 367), and Myctophidae ($n_{otoliths}$ = 413). All ODR time series had 197 10-y bins. **B**, Wavelet power spectra for proxies for sea surface temperature and primary productivity (10-y bins, n_{bins} =188). Time series detrended before wavelet analysis. Bold black lines are 95% confidence limits for wavelet power assuming a red-noise background with first-order autocorrelation. Thin, concave black line indicates cone of influence, below which wavelet power results are dubious. See Methods for details. Source data are provided as a Source Data file.

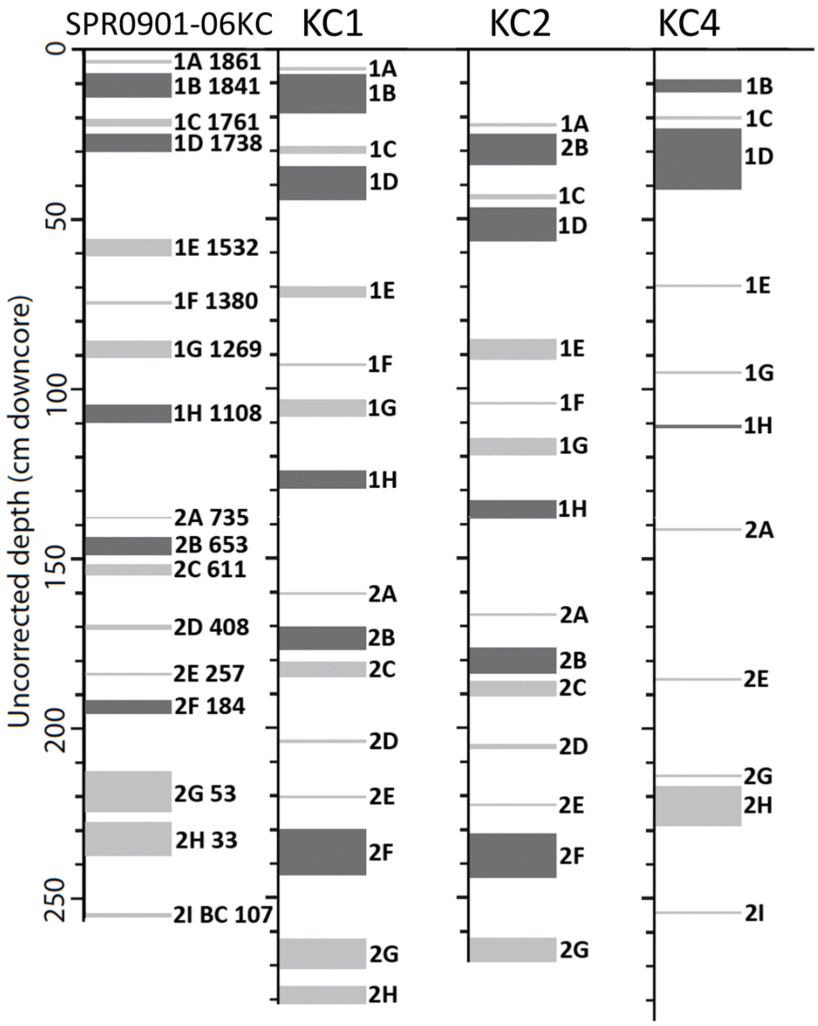

Supplementary Figure 5. Kasten core stratigraphy used to establish chronology. Uncorrected depth in centimeters of Kasten cores collected from the Santa Barbara Basin. Prominent instantaneous events, including gray and olive turbidites and gray flood layers, are indicated and labeled using the notation from Hendy *et al.* (ref. 1). Instantaneous events in core SPR0901-06KC were assigned calendar year dates by Hendy *et al.* (ref. 1) using terrestrial organic carbon and marine planktonic carbonate ^{14}C dates. To aid chronology development, instantaneous events were cross-dated between cores and with core SPR0901-06KC. The bottom approximately 50 cm of KC4 was not processed in this study.

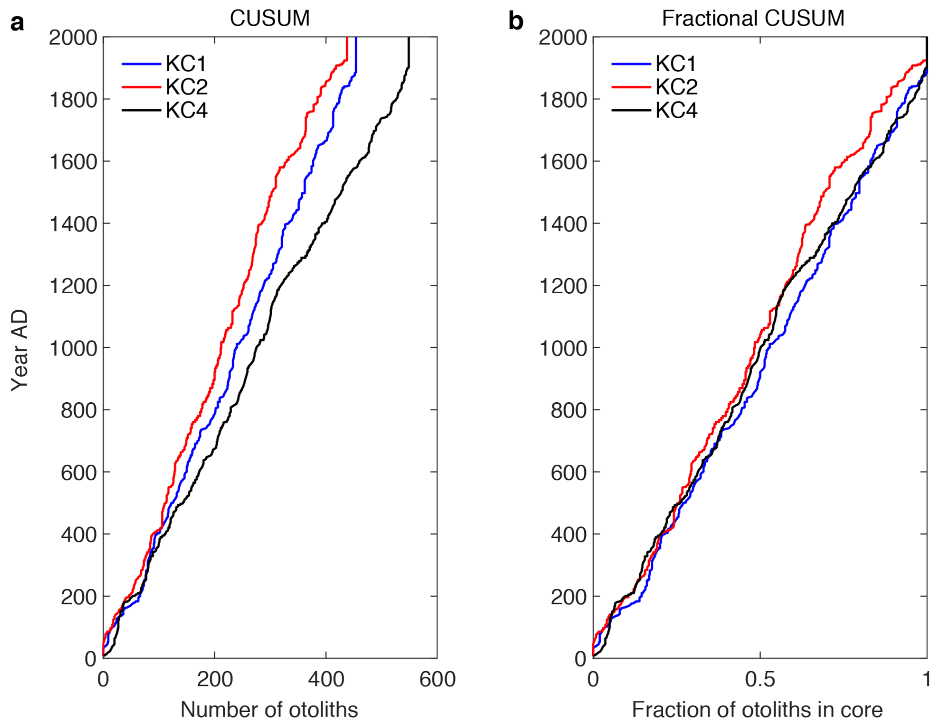

Supplementary Figure 6. Cumulative summations of otoliths in the Kasten cores. a, Cumulative summations for number of classified otoliths from core bottom to core top. The Kolmogorov-Smirnov (KS) test was used to test for differences between pairs of cumulative summation curves (Methods). All comparisons were significant at greater than $p = 0.001$ (two-tailed): $r_{KC1\text{-}KC2} = 0.12$, $r_{KC1\text{-}KC4} = 0.28$, and $r_{KC2\text{-}KC4} = 0.28$). **b**, Cumulative summations for fraction of otoliths in a core summed from core bottom (old) to core top (recent). The KS test was used to test for differences between pairs of cumulative summation curves. All probabilities are two-tailed: $r_{KC1\text{-}KC2} = 0.12$, $p_{KC1\text{-}KC2} = 0.002$, $r_{KC1\text{-}KC4} = 0.28$, $p_{KC1\text{-}KC4} = 0.087$, and $r_{KC2\text{-}KC4} = 0.28$, $p_{KC2\text{-}KC4} = 0.002$). Source data are provided as a Source Data file.

Supplementary Table 1. Classification of otoliths from the Santa Barbara Basin

Classification		Classification Method (Features)				
		Expert	RFA (Geo &EF)	DFA (Geo, EF & Elem)	Expert & RFA (Geo & EF)	Expert & DFA (Geo, EF & Elem)
Bathylagidae	$n_{otoliths}$	367	202	145	163	89
	% of classified	30.9	17.0	21.9	28.4	29.0
	% of identified	36.2	31.3	21.9	39.6	40.5
Myctophidae	$n_{otoliths}$	413	286	238	150	87
	% of classified	34.8	24.1	35.9	26.1	28.3
	% of identified	40.8	44.3	35.9	26.1	28.3
Engraulidae	$n_{otoliths}$	77	70	42	33	14
	% of classified	6.5	5.9	6.3	5.7	4.6
	% of identified	7.6	10.8	6.3	8.0	6.4
Clupeidae	$n_{otoliths}$	0	0	3	0	0
	% of classified	0	0	0.5	0	0
	% of identified	0	0	0.5	0	0
Merlucciidae	$n_{otoliths}$	110	31	67	44	22
	% of classified	9.3	2.6	10.1	7.7	7.2
	% of identified	10.9	4.8	10.1	10.7	10.0
Sebastidae	$n_{otoliths}$	46	57	168	22	8
	% of classified	3.9	4.8	25.3	3.8	2.6
	% of identified	4.5	8.8	25.3	5.3	3.6
Other	$n_{otoliths}$	175	542	0	162	87
	% of classified	14.7	45.6	0	28.2	28.3
Total	$n_{otoliths}$	1188	1188	663	574	307

Summary results of classification of otoliths from three Kasten and one box core from the Santa Barbara Basin spanning two millennia. 1524 otoliths were recovered from all cores. 336 otoliths were too altered to classify. Each classified otolith was assigned to one of six families or, if unidentifiable, ‘Other’. Classification methods were expert opinion (Expert), random forest analysis (RFA), discriminant function analysis (DFA) and combinations thereof. Feature types used for classification were geometric (Geo); elliptical Fourier (EF) and elemental composition (Elem). $n_{otoliths}$ is number of otoliths in a classification for each classification method. *% of classified* is the percent of otoliths of a classification in relation to the total number of classified (to family) otoliths. *% of identified* is the percent of otoliths of a classification in relation to the total number of identified (to family or Other) otoliths. Source data are provided as a Source Data file.

Supplementary References

1 Hendy, I. L., Dunn, L., Schimmelmann, A. & Pak, D. K. Resolving varve and radiocarbon chronology differences during the last 2000 years in the Santa Barbara Basin sedimentary record, California. *Quat. Int.* **310**, 155-168 (2013).

2 Jones, W. A. & Morales, M. M. *Catalog of otoliths of select fishes from the California Current System*, http://escholarship.org/uc/item/5m69146s (2014).
