# Supplementary material for: Mesopelagic fishes dominate otolith record of past two millennia in the Santa Barbara Basin
Source: Nat Commun. 2019 Oct 8;10:4564. doi: 10.1038/s41467-019-12600-z (PMC6783546; doi:10.1038/s41467-019-12600-z)
Supplement: Supplementary file 3 — Reporting Summary [file 41467_2019_12600_MOESM3_ESM.pdf]

nature research

Corresponding author(s): David M. Checkley, Jr.

Last updated by author(s): Sep 9, 2019

Reporting Summary

Nature Research wishes to improve the reproducibility of the work that we publish. This form provides structure for consistency and transparency in reporting. For further information on Nature Research policies, seeAuthors & Referees and theEditorial Policy Checklist .

Statistics

For all statistical analyses, confirm that the following items are present in the figure legend, table legend, main text, or Methods section.

n/a	Confirmed	
☐	☒	The exact sample size (*n*) for each experimental group/condition, given as a discrete number and unit of measurement
☐	☒	A statement on whether measurements were taken from distinct samples or whether the same sample was measured repeatedly
☐	☒	The statistical test(s) used AND whether they are one- or two-sided<br>*Only common tests should be described solely by name; describe more complex techniques in the Methods section.*
☐	☒	A description of all covariates tested
☐	☒	A description of any assumptions or corrections, such as tests of normality and adjustment for multiple comparisons
☐	☒	A full description of the statistical parameters including central tendency (e.g. means) or other basic estimates (e.g. regression coefficient) AND variation (e.g. standard deviation) or associated estimates of uncertainty (e.g. confidence intervals)
☐	☒	For null hypothesis testing, the test statistic (e.g. *F*, *t*, *r*) with confidence intervals, effect sizes, degrees of freedom and *P* value noted<br>*Give P values as exact values whenever suitable.*
☐	☐	For Bayesian analysis, information on the choice of priors and Markov chain Monte Carlo settings
☐	☐	For hierarchical and complex designs, identification of the appropriate level for tests and full reporting of outcomes
☐	☐	Estimates of effect sizes (e.g. Cohen's *d*, Pearson's *r*), indicating how they were calculated

Our web collection on statistics for biologists contains articles on many of the points above.

Software and code

Policy information about availability of computer code

Data collection	Only routine Matlab (R2019A) scripts ('m files') were used in this study.
Data analysis	Only routine Matlab (R2019A) scripts ('m files') and JMP Pro 14 statistical software were used in this study.

For manuscripts utilizing custom algorithms or software that are central to the research but not yet described in published literature, software must be made available to editors/reviewers. We strongly encourage code deposition in a community repository (e.g. GitHub). See the Nature Research guidelines for submitting code & software for further information.

Data

Policy information about availability of data

All manuscripts must include a data availability statement. This statement should provide the following information, where applicable:

- Accession codes, unique identifiers, or web links for publicly available datasets
- A list of figures that have associated raw data
- A description of any restrictions on data availability

Data Availability. The fossil otolith and environmental proxy data that support the findings of this study are available in UC San Diego Digital Collections (http://doi.org/10.6075/J0154FC9) (ref. 61). The source data underlying Figs 2, 3, 4, and 5, Tables 1 and 3, Supplementary Figs 3, 4 and 6 and Supplementary Table 1 are provided as a Source Data file.

Field-specific reporting

Please select the one below that is the best fit for your research. If you are not sure, read the appropriate sections before making your selection.

Ecological, evolutionary & environmental sciences study design

All studies must disclose on these points even when the disclosure is negative.

Study description	Otoliths (fish ear bones) were collected from Santa Barbara Basin sediment cores and used to construct time series of otolith deposition rate for families of fishes for analysis and comparison with climate proxies.
Research sample	A sample consists of otoliths extracted from a mass of sediment.
Sampling strategy	Four cores (three Kasten and one box) were collected from bottom of the Santa Barbara Basin. Each core was subsectioned to produce individual samples of known age and from which otoliths were extracted.
Data collection	WA Jones, with an undergraduate assistant (M Morales), removed otoliths from sediment samples microscopically. Said otoliths were analyzed to provide geometric and shape information elemental composition. Elemental composition was measured in the laboratory of Simon Thorrold at the Woods Hole Oceanographic Institution paid with funds from a California Sea Grant to DM Checkley, Jr.
Timing and spatial scale	The sediment cores spanned the past two millennia.
Data exclusions	No data were excluded from the analyses.
Reproducibility	Three Kasten cores, each spanning ~ two millennia, served as replicates that were compared (see Supplementary Figure 6).
Randomization	Not applicable.
Blinding	Expert classification of otoliths was done using masked (blinded) otoliths. Classification to family occurred only when two experts agreed, else an otolith was classified as 'other'. All elemental analyses were performed on masked (blinded) otoliths.

Did the study involve field work? ☒ Yes ☐ No

Field work, collection and transport

Field conditions	Samples were collected from the research vessel Melville. Surface conditions do not affect samples collected at the ocean bottom (~ 600 m depth). Ocean bottom conditions do not affect the underlying sediments collected by coring (upper ~ 2.5m).
Location	Santa Barbara Basin. See Fig 1. Box Core 1, Kasten Core 1 and Kasten Core 2 in this study were taken at station MV1012-ST46.9 (34° 17.228′N, 120° 02.135′ W). Kasten Core 4 was taken at station MV1012-ST46.2 (34° 13.700′ N, 120° 01.898′ W).
Access and import/export	Collection of sediment from the bottom of the Santa Barbara Basin for scientific purposes did not require permissions.
Disturbance	Disturbance was minimal (several hundred square centimeters of bottom disrupted by cores).

Reporting for specific materials, systems and methods

We require information from authors about some types of materials, experimental systems and methods used in many studies. Here, indicate whether each material, system or method listed is relevant to your study. If you are not sure if a list item applies to your research, read the appropriate section before selecting a response.

Materials & experimental systems

n/a	Involved in the study	
☐	☐	Antibodies
☐	☐	Eukaryotic cell lines
☐	☒	Palaeontology
☐	☐	Animals and other organisms
☐	☐	Human research participants
☐	☐	Clinical data

Methods

n/a	Involved in the study	
☐	☐	ChIP-seq
☐	☐	Flow cytometry
☐	☐	MRI-based neuroimaging

Antibodies

Antibodies used	*Describe all antibodies used in the study; as applicable, provide supplier name, catalog number, clone name, and lot number.*
Validation	*Describe the validation of each primary antibody for the species and application, noting any validation statements on the manufacturer's website, relevant citations, antibody profiles in online databases, or data provided in the manuscript.*

Eukaryotic cell lines

Policy information about cell lines

Cell line source(s)	*State the source of each cell line used.*
Authentication	*Describe the authentication procedures for each cell line used OR declare that none of the cell lines used were authenticated.*
Mycoplasma contamination	*Confirm that all cell lines tested negative for mycoplasma contamination OR describe the results of the testing for mycoplasma contamination OR declare that the cell lines were not tested for mycoplasma contamination.*
Commonly misidentified lines (See ICLAC register)	*Name any commonly misidentified cell lines used in the study and provide a rationale for their use.*

Palaeontology

Specimen provenance

No permits were required to obtain cores from Santa Barbara Basin sediments or the otoliths therein.

Specimen deposition

Laboratory of DM Checkley, Jr., Scripps Institution of Oceanography, La Jolla, CA 92037, USA.

Dating methods

Color photographs of each core were taken on the deck of the ship before subcoring. The three Kasten cores and one box core were subcored on deck with rectangular acrylic core liners ~ 76-cm long and 15 cm by 15 cm in cross section. Each Kasten core yielded four end-to-end subcores and the box core one subcore. All subcores, in acrylic liners, were placed into Hybar trilaminate membrane bags with oxygen absorbers, flushed with nitrogen, vacuum-sealed, and stored at 4°C. This storage method was successful in maintaining anoxic conditions within the sediments for several months until sample processing. One vertical slab ~ 2 cm thick was trimmed off the side of each subcore and X-radiographed at the Scripps Institution of Oceanography Geological Collections using a Geotek MSCL-XR core scanner. The core slabs were scanned at 1-mm intervals in a linear, non-rotational scan. Individual two-dimension images were combined to make the composite X-radiograph images.

X-radiographs and color photographs were used to develop a high-resolution chronology for each core. Several age models have been developed to assign dates to the SBB varved stratigraphy. The traditional age model relied on counting seasonal varve couplets42 and was used to establish a chronology for the top ~ 35 cm of BC1 in the present study. Analysis of 14C dates from planktonic foraminiferal carbonate and terrestrial-derived organic carbon from Kasten core SPR0901-06KC showed that accuracy of the traditional varve counting method decreased prior to ~1700 AD due to under-counting of varves14,43. Using 14C dates, a new SBB chronology was established from ~ 107 BC to 1700 AD14,43. We used this new SBB chronology to develop our Kasten core chronology, as follows. First, the major, near-instantaneous sedimentation events characterized and dated in Kasten core SPR0901-06KC were identified in our cores and assigned a single calendar date (Supplementary Fig. 5)14,42,43. The overall varve structure of each core was then cross-dated between our Kasten cores and core SPR0901-06KC to aid in the identification of layers formed rapidly during flood or turbidite events (Supplementary Fig. 1)15. The down-core chronology of each Kasten core was then corrected by excluding these near-instantaneous events. Finally, for each Kasten core, a series of linear regression equations between sequential, near-instantaneous events were developed to assign dates to the remaining stratigraphic structure14.

Varves of BC1 were counted from 2009 back to 1871 AD. Visual cross-dating agreed well with the chronology of box cores dated previously42. The sediment chronology prior to 1871 for BC1 and for each entire Kasten core was resolved every 0.5 cm, exclusive of near-instantaneous event layers, by cross-dating as described above)14,43, extending the chronology of BC1 back to 1836 AD and the Kasten cores back to 8-53 AD. The bottom ~ 50 cm of KC4 was not processed due to low confidence in the stratigraphic chronology (Supplementary Fig. 5).

Each 15-cm x 13-cm cross section subcore was cut transversely every 0.5 cm, if no near-instantaneous event layer occurred, to create transverse sections (97.5 cm3). Near-instantaneous event layers were added to respective transverse sections, in which cases the volume exceeded 97.5 cm2. Transverse sections were stored frozen at -80°C until further processing. Each transverse section represented a time interval of ~2 to 8 years. The date assigned each transverse section was the average of the dates of its upper and lower surfaces.

☒ Tick this box to confirm that the raw and calibrated dates are available in the paper or in Supplementary Information.

Animals and other organisms

Policy information about studies involving animals; ARRIVE guidelines recommended for reporting animal research

Laboratory animals	*For laboratory animals, report species, strain, sex and age OR state that the study did not involve laboratory animals.*
Wild animals	*Provide details on animals observed in or captured in the field; report species, sex and age where possible. Describe how animals were caught and transported and what happened to captive animals after the study (if killed, explain why and describe method; if released, say where and when) OR state that the study did not involve wild animals.*
Field-collected samples	*For laboratory work with field-collected samples, describe all relevant parameters such as housing, maintenance, temperature, photoperiod and end-of-experiment protocol OR state that the study did not involve samples collected from the field.*

Ethics oversight | *Identify the organization(s) that approved or provided guidance on the study protocol, OR state that no ethical approval or guidance was required and explain why not.*

Note that full information on the approval of the study protocol must also be provided in the manuscript.

Human research participants

Policy information about studies involving human research participants

Population characteristics | *Describe the covariate-relevant population characteristics of the human research participants (e.g. age, gender, genotypic information, past and current diagnosis and treatment categories). If you filled out the behavioural & social sciences study design questions and have nothing to add here, write "See above."*

Recruitment | *Describe how participants were recruited. Outline any potential self-selection bias or other biases that may be present and how these are likely to impact results.*

Ethics oversight | *Identify the organization(s) that approved the study protocol.*

Note that full information on the approval of the study protocol must also be provided in the manuscript.

Clinical data

Policy information about clinical studies

All manuscripts should comply with the ICMJEguidelines for publication of clinical research and a completedCONSORT checklist must be included with all submissions.

Clinical trial registration | *Provide the trial registration number from ClinicalTrials.gov or an equivalent agency.*

Study protocol | *Note where the full trial protocol can be accessed OR if not available, explain why.*

Data collection | *Describe the settings and locales of data collection, noting the time periods of recruitment and data collection.*

Outcomes | *Describe how you pre-defined primary and secondary outcome measures and how you assessed these measures.*

ChIP-seq

Data deposition

☐ Confirm that both raw and final processed data have been deposited in a public database such as GEO.

☐ Confirm that you have deposited or provided access to graph files (e.g. BED files) for the called peaks.

Data access links
May remain private before publication. | *For "Initial submission" or "Revised version" documents, provide reviewer access links. For your "Final submission" document, provide a link to the deposited data.*

Files in database submission | *Provide a list of all files available in the database submission.*

Genome browser session
(e.g. UCSC) | *Provide a link to an anonymized genome browser session for "Initial submission" and "Revised version" documents only, to enable peer review. Write "no longer applicable" for "Final submission" documents.*

Methodology

Replicates | *Describe the experimental replicates, specifying number, type and replicate agreement.*

Sequencing depth | *Describe the sequencing depth for each experiment, providing the total number of reads, uniquely mapped reads, length of reads and whether they were paired- or single-end.*

Antibodies | *Describe the antibodies used for the ChIP-seq experiments; as applicable, provide supplier name, catalog number, clone name, and lot number.*

Peak calling parameters | *Specify the command line program and parameters used for read mapping and peak calling, including the ChIP, control and index files used.*

Data quality | *Describe the methods used to ensure data quality in full detail, including how many peaks are at FDR 5% and above 5-fold enrichment.*

Software | *Describe the software used to collect and analyze the ChIP-seq data. For custom code that has been deposited into a community repository, provide accession details.*

Flow Cytometry

Plots

Confirm that:

- ☐ The axis labels state the marker and fluorochrome used (e.g. CD4-FITC).
- ☐ The axis scales are clearly visible. Include numbers along axes only for bottom left plot of group (a 'group' is an analysis of identical markers).
- ☐ All plots are contour plots with outliers or pseudocolor plots.
- ☐ A numerical value for number of cells or percentage (with statistics) is provided.

Methodology

Sample preparation	*Describe the sample preparation, detailing the biological source of the cells and any tissue processing steps used.*
Instrument	*Identify the instrument used for data collection, specifying make and model number.*
Software	*Describe the software used to collect and analyze the flow cytometry data. For custom code that has been deposited into a community repository, provide accession details.*
Cell population abundance	*Describe the abundance of the relevant cell populations within post-sort fractions, providing details on the purity of the samples and how it was determined.*
Gating strategy	*Describe the gating strategy used for all relevant experiments, specifying the preliminary FSC/SSC gates of the starting cell population, indicating where boundaries between "positive" and "negative" staining cell populations are defined.*

☐ Tick this box to confirm that a figure exemplifying the gating strategy is provided in the Supplementary Information.

Magnetic resonance imaging

Experimental design

Design type	*Indicate task or resting state; event-related or block design.*
Design specifications	*Specify the number of blocks, trials or experimental units per session and/or subject, and specify the length of each trial or block (if trials are blocked) and interval between trials.*
Behavioral performance measures	*State number and/or type of variables recorded (e.g. correct button press, response time) and what statistics were used to establish that the subjects were performing the task as expected (e.g. mean, range, and/or standard deviation across subjects).*

Acquisition

Imaging type(s)	*Specify: functional, structural, diffusion, perfusion.*
Field strength	*Specify in Tesla*
Sequence & imaging parameters	*Specify the pulse sequence type (gradient echo, spin echo, etc.), imaging type (EPI, spiral, etc.), field of view, matrix size, slice thickness, orientation and TE/TR/flip angle.*
Area of acquisition	*State whether a whole brain scan was used OR define the area of acquisition, describing how the region was determined.*

Diffusion MRI ☐ Used ☐ Not used

Preprocessing

Preprocessing software	*Provide detail on software version and revision number and on specific parameters (model/functions, brain extraction, segmentation, smoothing kernel size, etc.).*
Normalization	*If data were normalized/standardized, describe the approach(es): specify linear or non-linear and define image types used for transformation OR indicate that data were not normalized and explain rationale for lack of normalization.*
Normalization template	*Describe the template used for normalization/transformation, specifying subject space or group standardized space (e.g. original Talairach, MNI305, ICBM152) OR indicate that the data were not normalized.*
Noise and artifact removal	*Describe your procedure(s) for artifact and structured noise removal, specifying motion parameters, tissue signals and physiological signals (heart rate, respiration).*
Volume censoring	*Define your software and/or method and criteria for volume censoring, and state the extent of such censoring.*

Statistical modeling & inference

Model type and settings

Specify type (mass univariate, multivariate, RSA, predictive, etc.) and describe essential details of the model at the first and second levels (e.g. fixed, random or mixed effects; drift or auto-correlation).

Effect(s) tested

Define precise effect in terms of the task or stimulus conditions instead of psychological concepts and indicate whether ANOVA or factorial designs were used.

Specify type of analysis: ☐ Whole brain ☐ ROI-based ☐ Both

Statistic type for inference
(See Eklund et al. 2016)

Specify voxel-wise or cluster-wise and report all relevant parameters for cluster-wise methods.

Correction

Describe the type of correction and how it is obtained for multiple comparisons (e.g. FWE, FDR, permutation or Monte Carlo).

Models & analysis

n/a	Involved in the study
☐	☐ Functional and/or effective connectivity
☐	☐ Graph analysis
☐	☐ Multivariate modeling or predictive analysis

Functional and/or effective connectivity

Report the measures of dependence used and the model details (e.g. Pearson correlation, partial correlation, mutual information).

Graph analysis

Report the dependent variable and connectivity measure, specifying weighted graph or binarized graph, subject- or group-level, and the global and/or node summaries used (e.g. clustering coefficient, efficiency, etc.).

Multivariate modeling and predictive analysis

Specify independent variables, features extraction and dimension reduction, model, training and evaluation metrics.
